# Multifaceted Evaluation of *Paliurus spina-christi* Mill. Plant, Seeds, and Oils: Hydrogen Cyanide, Phytochemical, Bioactive, and Antimicrobial Insights from Türkiye Ecotypes

**DOI:** 10.3390/molecules31010087

**Published:** 2025-12-25

**Authors:** Rabiya Safiye Çelebi, Erman Duman

**Affiliations:** Department of Food Engineering, Faculty of Engineering, Afyon Kocatepe University, 03200 Afyonkarahisar, Türkiye; rabiaozdemir@gmail.com

**Keywords:** *Paliurus spina-christi*, seed oils, toxicological, chemical, phytochemicals, antimicrobial

## Abstract

This investigation comparatively examined the toxicological, chemical, phytochemical, and antimicrobial characteristics of *Paliurus spina-christi* Mill. seeds and their oils collected over two consecutive years from four distinct locations in Türkiye. In the seeds, HCN levels ranged from 1.11 to 1.43 g/kg and total phenolics from 6.84 to 15.48 mg GAE/g, while quinic, gallic, protocatechlic, and tannic acids, along with cosmosiin, were identified as the main phenolics in the phenolic profile. In the seed oils, α-tocopherol content ranged from 2178.5 to 2528.4 mg/kg, total phenolics from 96.99 to 118.87 mg GAE/g, and antioxidant activity from 0.147 to 0.150 mg TE/g. β-Sitosterol predominated among sterols (61.51–66.51%). Macrominerals P, S, K, and Ca and microminerals Si, Pt, Pd, Ge, and Sn were present in notable amounts. An antimicrobial activity test revealed the bacteriostatic effects of the seed oils. In conclusion, this study elucidates the toxicological, chemical, phytochemical, and antimicrobial attributes of *P. spina-christi* seeds and oils, showing that the proportions of the identified bioactive components varied according to harvest year and location. Based on this data, it is recommended that further research is conducted in the future regarding the potential use of *P. spina-christi* seeds/seed oil for human nutrition, in terms of standardization, bioavailability, and clinical validation.

## 1. Introduction

The Mediterranean basin, West Asia, and Southern Europe are home to *Paliurus spina-christi Mill.*, a deciduous, prickly shrub or small tree that is a member of the Rhamnaceae family. It is widely found in the Mediterranean, Southern Marmara, Aegean, and Central Anatolia of Türkiye, where it is known locally as “karaçalı” or “Christ’s thorn” [1,2]. Within the Rhamnaceae family’s five genera (*Paliurus*, *Zizyphus*, *Rhamnus*, *Frangula*, *Sageretia*), the genus *Paliurus* Tourn. ex Miller comprises five species globally: *P. spina-christi*, *P. Orientalis*, *P. hemsleyanus*, *P. hirsutus*, and *P. ramosissimus*. Among them, only *P. spina-christi* exists in Türkiye. In traditional medicine, *P. spina-christi*’s fruit, leaves, and bark have historically been used as diuretics, anti-inflammatory agents, cholesterol-lowering agents, tonics, wound-healing agents, antidiabetic agents, and anti-rheumatic agents. Furthermore, the dried fruits are incorporated into food supplements or tea mixtures in certain regions [3,4,5,6,7,8].

Little research has been conducted on the physicochemical composition of *P. spina-christi* seed oils. The available literature indicates that the seeds of this plant contain 20% fixed oil, with fatty acids, such as palmitic acid (7.7–8%), stearic acid (3.5–10.3%), oleic acid (35.3–36.9%), and linoleic acid (38.2–43.9%), making up the majority of the oil. In terms of sterol composition, phytosterols such as β-sitosterol (66%), stigmasterol (13%), and campesterol (11%) have been reported. These components of *P. spina-christi* oils, which are stated to be rich in phenolic and flavonoid contents, have been emphasized as being important in terms of both the nutritional value and functional activity of the oil [9,10]. Various parts of *P. spina-christi* (branches, leaves, flowers, bark, and fruits) have been phytochemically investigated and were found to contain flavonoids such as catechin, rutin, isoquercetin, quercetin-3-rutinoside-7-rhamnoside, kaempferol-3-glucoside, epigallocatechin, gallocatechin, and catechol, as well as tannins, amino acids, and alkaloids. In addition, the presence of minerals, including sodium, calcium, magnesium, phosphorus, and zinc, has been reported [11,12,13]. Accordingly, fixed oils of medicinal and aromatic plants, such as *P. spina-christi*, are considered potential functional components [14]. Studies on leaf and seed extracts of *P. spina-christi* have reported that, in a food context, these extracts possess high phenolic content and strong antioxidant activity [15,16]. From a medical perspective, it has been suggested that these extracts are promising for diabetes treatment by activating insulin signaling pathways [17].

In this context, the HCN content, total phenolic content, and phenolic compound composition of *P. spina-christi* seeds collected from four different ecological locations (Konya–Akşehir, Burdur–Bucak, Muğla–Köyceğiz, and Balıkesir–Sındırgı) in Türkiye in two different years (2023 and 2024), as well as the total phenolic content, antioxidant activity, vitamin E content, mineral content, sterol composition, and antimicrobial properties of the seed oils were systematically investigated. The purpose of this investigation is to close the data gap in the literature through a multi-location and two-year investigation, and to reveal the chemical, toxicological, phytochemical, and antimicrobial properties of *P. spina-christi* seeds and oils based on the results obtained.

## 2. Results and Discussion

### 2.1. HCN Content of Seeds

The HCN contents determined in *P. spina-christi* seeds are presented in Table 1. In the first and second harvest years, the HCN contents showed statistically significant differences between locations (*p* < 0.05). In the first harvest year, HCN values were measured in the range of 1.13–1.87 g/kg, while in the second harvest, these values were found in the range of 0.99–1.29 g/kg. According to the annual average values, the highest HCN content was determined at the Konya–Akşehir location (1.43 g/kg), and the lowest at the Muğla–Köyceğiz and Balıkesir–Sındırgı locations (1.11 g/kg). A decreasing trend in HCN content was observed in all species between harvest years.

Esfahani et al. (2023) reported that extracts from *P. spina-christi* fruits did not contain saponins or cyanogenic glycosides but were rich in alkaloids, tannins, steroids, and flavonoids [17]. No other studies directly examining the HCN content of *P. spina-christi* seeds were identified. Therefore, a study on the toxic effects of *Rhamnus alaternus*, a related species from the same family, was examined. The study concluded that this plant has strong antibacterial, antioxidant, and antidiabetic effects and is toxic when overused [18].

International safety assessments for hydrogen cyanide (HCN) levels in foods recommend a maximum limit of 0.09 mg HCN/kg body weight [19]. Similarly, the European Food Safety Authority established a threshold value for acute toxicity at 20 μg/kg body weight, emphasizing that even low levels of exposure may be significant, particularly in sensitive individuals [20]. It was determined that all HCN values obtained in this study were well below the mentioned limits, indicating that consumption did not pose any risks.

### 2.2. Total Phenolic Contents of Seeds

As can be seen in Table 2, the total phenolic contents (mg GAE/g) determined in *P. spina-christi* seeds showed statistically substantial variations among the first and second harvest years (*p* < 0.05). According to the first harvest year data, the highest phenolic content was determined at the Konya–Akşehir location (17.52 mg GAE/g), while the lowest value was determined at the Burdur–Bucak location (6.10 mg GAE/g). When the second harvest year’s values were examined, the phenolic total contents were measured as 13.44 mg GAE/g at the Konya–Akşehir location and 7.05 mg GAE/g in the Muğla–Köyceğiz location, and it was observed that there was a decrease at both locations compared to the previous year. In contrast, a value of 7.59 mg GAE/g was measured at the Burdur–Bucak location and 10.18 mg GAE/g at the Balıkesir–Sındırgı location, with an increase compared to the previous year. Considering the annual average values, the greatest phenolic total content was observed at the Konya–Akşehir location (15.48 mg GAE/g) and the lowest at the Burdur–Bucak location (6.84 mg GAE/g).

Literature reports have examined the total phenol contents in the leaves and seeds of *P. spina-christi* plants. The total phenol (TP) content was determined as 16.98 mg GAE/g in the methanol and hexane extracts of the leaves and as 1.10 mg GAE/g in the methanol extract of the seeds [15]. Takım (2021) determined the total phenolic content as 22.10 mg GAE/g following water extraction of *P. spina-christi* fruits [13]. In another study, the TP content for methanol extract was determined as 109.54 mg GAE/g extract [21]. Therefore, it can be seen that the seed total phenolic contents obtained in our study were higher than those reported for *P. spina-christi* seeds in the literature, but lower than the values reported for fruits.

### 2.3. Phenolic Compound Composition of Seeds

The phenolic compound composition of *P. spina-christi* seeds is presented in Table 3. Significant differences were determined between the first and second harvest years. In the analyses, a total of 56 phenolic compounds were screened, 18 of which were detected in at least one location or year. The most dominant compounds included quinic acid, gallic acid, protocatechuic acid, tannic acid, and cosmosiin. In addition, flavonoid compounds such as genistin, amentoflavone, and acacetin were detected in low amounts.

In the samples from the first harvest year, quinic acid (0.192–0.655 mg/g) and gallic acid (0.131–0.329 mg/g) were detected at high concentrations. Protocatechuic acid values ranged from 0.022 to 0.032 mg/g, with the greatest score measured at the Konya–Akşehir location. Tannic acid contents were determined to be between 0.032 and 0.348 mg/g, with a significant accumulation observed especially at the Konya–Akşehir location (0.348 mg/g). Cosmosiin, from the flavonoid group, was measured at a level of 0.025–0.078 mg/g, reaching the highest value at the Balıkesir–Sındırgı location. Fumaric acid (0.114 mg/g), isoquercitrin (0.022 mg/g), hesperidin (0.022 mg/g), daidzein (0.008 mg/g), luteolin (0.005 mg/g), apigenin (0.004 mg/g), chrysin (0.004 mg/g), and acacetin (0.022 mg/g) were detected only at the Konya–Akşehir location. Genistin, naringenin, and amentoflavone were determined at the Konya–Akşehir (0.024, 0.007, 0.049 mg/g, respectively) and Burdur–Bucak (0.010, 0.005, 0.005 mg/g, respectively) locations.

The samples from the second harvest year typically showed reductions in the phenolic chemical contents. While quinic acid levels were found to be highest at the Konya–Akşehir location at 0.725 mg/g, this value varied between 0.168 and 0.455 mg/g at other locations. With the exception of the Muğla–Köyceğiz location, an increase in gallic acid was observed at the other locations, with contents varying between 0.169 and 0.238 mg/g. Protocatechuic acid (0.014–0.038 mg/g), tannic acid (0.048–0.056 mg/g), and cosmosiin (0.022–0.065 mg/g) were also found at all locations in the second harvest year. Salicylic acid (0.019 mg/g), isoquercitin (0.050 mg/g), hesperidin (0.016 mg/g), amentoflavone (0.010 mg/g), genistin (0.005 mg/g), and acacetin (0.007 mg/g) were seen only at the Konya–Akşehir location. Naringenin was found at the Konya–Akşehir (0.003 mg/g) and Burdur–Bucak (0.003 mg/g) locations, while rutin was observed at the Konya–Akşehir (0.053 mg/g) and Balıkesir–Sındırgı (0.028 mg/g) locations. Chlorogenic acid appeared only at the Burdur–Bucak site (0.014 mg/g).

Examining the correlation between the phenolic compositions and contents of *P. spina-christi* seeds, as seen in Table 2 and Table 3, both the total phenolic contents of the seeds and the number/contents of detected phenolic components increased.

A study examining 53 phenolic compounds in *P. spina-christi* fruit extracts obtained using different extraction methods identified 30 phenolic components in methanol extracts. The most abundant components were rutin (66,778.5 μg/g), catechin (43,696.5 μg/g), hesperidin (36,698.4 μg/g), epigallocatechin (5749 μg/g), epicatechin (8024.4 μg/g), vanillic acid (9046.3 μg/g), malic acid (17,536.8 μg/g), quinic acid (28,388.3 μg/g), and nicotiflorin (2081.0 μg/g) [11]. Another study employing LC-MS analysis on samples from different parts of the *P. spina-christi* plant reported that methanol and water extracts of branches contained 39 and 67 compounds, respectively, with quercetin and epigallocatechin derivatives being the most prominent. The most prevalent chemicals found in fruit extracts were sugar and quercetin derivatives, with 24 and 20 compounds found in methanol and water extracts, respectively. The methanol extract, containing 54 chemicals, had the greatest kaempferol content among the leaf extracts. Conversely, 78 compound elements were observed in the water extract, with kaempferol and quercetin compounds predominating [16]. In our study, the phenolic compound composition of the seeds, compared to the phenolic contents reported in the literature for the fruits, branches, and leaves of *P. spina-christi*, was found to be lower in both diversity and content.

### 2.4. α-Tocopherol Contents of Seed Oils

Table 4 reveals statistically notable variations (*p* < 0.05) in the total α-tocopherol contents of *P. spina-christi* seed oils between the first and second harvest years. In the first harvest year, the average α-tocopherol content was 1177.2 mg/kg at the Konya–Akşehir location, 1262.8 mg/kg at the Burdur–Bucak location, 1320.5 mg/kg at the Muğla–Köyceğiz location, and 1285.6 mg/kg at the Balıkesir–Sındırgı location. The Muğla–Köyceğiz location exhibited the highest α-tocopherol content, while the Konya–Akşehir location showed the lowest.

The second harvest year data indicate a significant increase in α-tocopherol levels across all locations. The Burdur–Bucak location exhibited the highest value (3794 mg/kg), followed by Konya–Akşehir (3698 mg/kg), Balıkesir–Sındırgı (3361.5 mg/kg), and Muğla–Köyceğiz (3036.5 mg/kg). Examining the average values by year, the total α-tocopherol contents varied between 2178.5 and 2528.4 mg/kg.

In a study examining the tocopherol contents of 48 plants, including *P. spina-christi*, the total tocopherol content of *P. spina-christi* seed oil was determined to be 236 mg/kg, with α-tocopherol comprising 87.7% [22]. Since there are few resources in the literature regarding *P. spina-christi*, a study conducted on *Z. lotus* kernel oil, which is from the same family as *P. spina-christi*, was examined, and it was observed that it did not contain α-tocopherol, but it contained the highest contents (130.47 and 10.60 mg/100 g, respectively) of β-tocopherol and δ-tocopherol [23]. In our study, the α-tocopherol levels determined in *P. spina-christi* seed oils were higher compared to the values obtained in the other studies.

### 2.5. Total Phenolic Contents of Seed Oil

The total phenolic contents (mg GAE/g) of *P. spina-christi* seed oils showed statistically notable variations between the first and second harvest years (*p* < 0.05) (Table 5). According to the first harvest year data, the highest total phenolic content was determined at the Burdur–Bucak (146.09 mg GAE/g) and Muğla–Köyceğiz (145.41 mg GAE/g) locations.

A decreasing trend in the total phenolic contents was generally observed in the second harvest year values. However, a significant increase was detected at the Balıkesir–Sındırgı location (134.15 mg GAE/g). The values at the other locations varied between 84.01 and 92.33 mg GAE/g. The highest year average was determined at the Muğla–Köyceğiz location (118.87 mg GAE/g), and the smallest average was determined at the Konya–Akşehir location (96.99 mg GAE/g). In our study, the α-tocopherol levels determined in the *P. spina-christi* seed oils were higher compared to the measurements observed in the other studies. Analyses of total phenolic content in *P. spina-christi* seeds and seed oils in our study showed that the phenolic levels in the oil fraction were higher compared to those in the seed material.

No studies on the total phenolic contents of *P. spina-christi* seed oils have been found in the literature. The total phenolic contents obtained from *P. spina-christi* fruits were determined as 61.24, 2.44, 85.13, 17.81, and 36.49 mg GAE/g with ethanol, n-hexane, chloroform, ethyl acetate, and aqueous ethanol extracts, respectively [5]. Zengin et al.’s study on the phenolic contents in extracts of the leaves, stems, and fruits of *P. spina-christi* using different solvents showed that the methanol extract of the fruits had the highest total phenolic content (75.91 mg GAE/g), followed by the water, ethyl acetate, n-hexane, and dichloromethane extracts [16]. Comparing the total phenolic content values of seed oil obtained in our study with the total phenolic contents reported for the fruit, leaf, and stem portions of *P. spina-christi* in the literature, it was found that the phenolic levels of the seeds were higher, especially compared to the fruits.

### 2.6. Antioxidant Activity of Seed Oils

As presented in Table 6, the antioxidant activity of *P. spina-christi* seed oils was evaluated in mg TE/g, and no statistically notable variations were detected among the locations or harvest years (*p* > 0.05). Among the first harvest year values, the highest antioxidant capacity was seen at the Konya–Akşehir location (0.151 mg TE/g), followed by the Burdur–Bucak (0.150 mg TE/g), Muğla–Köyceğiz (0.149 mg TE/g), and Balıkesir–Sındırgı (0.146 mg TE/g) locations.

The values among all locations are quite close and generally show a slight downward trend in the second harvest year. The highest value was determined at Konya–Akşehir (0.150 mg TE/g), and the smallest value was determined at Muğla–Köyceğiz (0.146 mg TE/g). Considering the annual averages, Konya–Akşehir (0.150 mg TE/g) showed the greatest value, whereas the Muğla–Köyceğiz and Balıkesir–Sındırgı locations (0.147 mg TE/g) showed the smallest value. These findings reveal that although there is no notable variation among the locations in terms of antioxidant capacity, the Konya–Akşehir location consistently showed higher activity. Furthermore, Table 4 and Table 6 demonstrate the correlation between the α-tocopherol and antioxidant activity of the *P. spina-christi* seed oils.

Previous research on water extracts of *P. spina-christi* fruits reported an antioxidant value of 23.10 [24]. Another study utilizing branch, leaf, and fruit extracts of *P. spina-christi* (hexane, ethyl acetate, ethanol, and chloroform) indicated high ABTS and DPPH radical scavenging antioxidant activity for all extracts except hexane [5]. Similarly, methanol, chloroform, and ethyl acetate extracts of *P. spina-christi* were reported to possess high antioxidant activity against the DPPH radical [25]. Methanol extracts of *P. spina-christi* fruits were also reported to exhibit strong DPPH radical scavenging activity [21]. Furthermore, *P. spina-christi* had the highest antioxidant activity, according to separate research that employed methanol extracts of 28 distinct plants in an effort to identify new possible natural antioxidant sources [26]. A study measuring the antioxidant capacities of extracts obtained from different parts of the *P. spina-christi* plant with different solvents determined that methanol extracts exhibited the highest antioxidant capacity in all parts (909.88 mg TE/g in stems, 245.59 mg TE/g in fruits, and 480.10 mg TE/g in leaves) [16]. Comparing the antioxidant activity values of *P. spina-christi* seed oils obtained in our study with the antioxidant capacity levels reported for the fruit, leaf, and stem tissues of the plant in the literature, the antioxidant property of seed oils was determined to be lower compared to other botanical parts.

### 2.7. Sterol Compositions of Seed Oils

Evaluating the sterol composition detected in *P. spina-christi* seed oils, β-sitosterol was found to be the dominant sterol component among all locations and harvest periods (Table 7). The average values of the β-sitosterol ratio according to location varied between 61.51% and 66.51%, with the highest value determined at the Konya–Akşehir location (66.51%) and the lowest value determined at the Muğla–Köyceğiz location (61.51%). This finding reveals that β-sitosterol constitutes approximately two-thirds of the total sterol fraction of seed oil. Furthermore, the average campesterol ratio of seed oils varied between 6.69 and 7.26%, and the highest ratio was at the Konya–Akşehir location, while the stigmasterol ratio varied between 9.86% and 11.70% and the highest value was at the Burdur–Bucak location. The Δ5-avenasterol ratio varied between 6.73 and 7.81% and was found in higher concentrations, especially at the Muğla–Köyceğiz location. Minor sterols, such as Δ7-stigmasterol, Δ7-avenasterol, brassicasterol, and cholesterol, were generally not detected in the first harvest year, while they were determined at very low rates (0.01–0.45%) in the second harvest year. Fluctuations were observed between harvest years in terms of certain sterol components; for example, the β-sitosterol ratio was higher in the first harvest, while a partial decrease trend was observed in the second harvest. Statistical analyses revealed that while many components (such as brassicasterol, campesterol, and cholesterol) did not significantly differ between species in the second year, the differences between all sterol components in the first year were statistically notable (*p* < 0.05).

The sterol composition obtained in this study is significantly similar to previous studies on *P. spina-christi* and related species in the literature. A study from Georgia reported that *P. spina-christi* seed oil contained 66% β-sitosterol, 13% stigmasterol, and 11% campesterol [10]. Limited resources regarding *P. spina-christi* were found in the literature. In a study examining the sterol composition of *Zizyphus lotus* seed oils, a species belonging to the same family as *P. spina-christi*, the main sterols were determined to be Δ7-campesterol (147.82 mg/100 g oil) and β-sitosterol (82.10 mg/100 g oil). In the same study, the contents of campesterol and stigmasterol were determined as 31.89 mg/100 g oil and 16.38 mg/100 g oil, respectively [23]. Comparing the sterol composition of *P. spina-christi* seed oils obtained in our study with the data reported in other studies, it was determined that the β-sitosterol levels were similar, while the contents of campesterol and stigmasterol were lower. When compared with other species, all sterol components were determined to be at lower levels.

### 2.8. Mineral Composition of Seed Oils

A total of 40 mineral substances, 6 of which were macrominerals and 34 of which were microminerals, were analyzed in *P. spina-christi* seed oils. As presented in Table 8, phosphorus (P) concentrations ranged from 304.8 to 388.25 ppm (average of 317.42 to 355.08 ppm), sulfur (S) ranged from 329.08 to 354.58 ppm (average of 331.17 to 347.92 ppm), potassium (K) ranged from 54.42 to 65.11 ppm (average of 55.28 to 60.79 ppm), and calcium (Ca) ranged from 35.83 to 92.49 ppm (average of 48.66 to 66.44 ppm). These were identified as the dominant macrominerals across all locations. Magnesium (Mg) concentrations varied between 19.05 and 49.10 ppm (average of 26.53 to 35.99 ppm), while sodium (Na) concentrations ranged from 23.72 to 50.38 ppm (average of 28.11 to 40.12 ppm). Statistical analysis revealed notable variations (*p* < 0.05) in macromineral contents between locations for both harvest years, with the exception of potassium (K). The Balıkesir–Sındırgı location exhibited the highest average values for Mg, K, and P, while the Burdur–Bucak location showed the highest average values for Na and S. The Muğla–Köyceğiz location presented the highest value for Ca.

The micromineral contents of *P. spina-christi* seed oils are shown in Table 9. The analysis results show that the microminerals with the highest concentrations were Si (avg. of 200.51–241.61 ppm), Pt (avg. of 79.04–88.31 ppm), Pd (avg. of 37.26–41.87 ppm), Ge (avg. of 34.63–43.85 ppm), Sn (avg. of 30.14–37.16 ppm), Ga (avg. of 29.35–34.05 ppm), Cr (avg. of 12.72–20.28 ppm), and Fe (avg. of 3.90–22.92 ppm). Except for some mineral substances (such as V, Nb, Ti, Cd, Li, Ag, Ge, and Ti), the interaction between the location and harvest year factors was measured to be statistically notable (*p* < 0.05). Generally, the highest values for these minerals were found at the Balıkesir–Sındırgı and Muğla–Köyceğiz locations. Copper (Cu) was not detected at any of the examined locations.

Mineral substance analyses carried out on *P. spina-christi* samples collected from the Mediterranean region for one year revealed average values of 18.9, 2.85, 1.77, 19.1, 2.83, and 1.24 g/kg for S, Mg, Ca, K, P, and N as macro-elements, respectively [27]. A study examining mineral composition by combining samples from various parts of the *P. spina-christi* plant (branches, leaves, and fruits) obtained from the Eastern Mediterranean region found the highest concentrations of Ca, K, Mg, Na, Zn, and Al [28]. Takım (2021) analyzed the mineral contents in *P. spina-christi* fruits and reported the highest concentrations of K (2017 mg/kg), Ca (790.8 mg/kg), Mg (275.03 mg/kg), P (254.13 mg/kg), Na (193.96 mg/kg), and Fe (43.71 mg/kg) [13]. In a study on *Zizyphus lotus* seed oils, which belong to the same family as *P. spina-christi*, the highest rates of magnesium (153.92 mg/100 g), calcium (110.58 mg/100 g), and potassium (92.41 mg/100 g) minerals were determined in mineral substance analyses [23]. No information was found in the literature regarding the mineral substance composition of *P. spina-christi* seed oils. Comparing the mineral substance composition of *P. spina-christi* seed oils obtained in our study with the values reported for *P. spina-christi* fruits in the literature showed that they were at lower levels. In addition, compared with the mineral substance contents reported in the seed oils of other species, the mineral levels in *P. spina-christi* seed oil were lower.

### 2.9. Antimicrobial Activities of Seed Oils

The antimicrobial activity of *P. spina-christi* seed oils was assessed using the agar diffusion method for bacteria and the agar well method for mold species. The results indicate a significant inhibitory effect against bacterial species, while no antifungal activity was observed against the tested mold species (Table 10). The antimicrobial activity results of *P. spina-christi* seed oils show statistically significant differences according to location and harvest year (*p* < 0.05).

The highest antibacterial effect against *Escherichia coli* was determined in the first harvest year at the Konya–Akşehir location with a zone diameter of 36 mm. This value was significantly higher compared to other species but decreased to 10 mm in the second harvest year. Zone diameters of 32 mm and 23 mm were measured against *E. coli* at the Burdur–Bucak location during Year 1 and Year 2, respectively; 14 mm and 22 mm at the Muğla–Köyceğiz location in Year 1 and Year 2; and 28 mm and 24 mm at the Balıkesir–Sındırgı location across Year 1 and Year 2, respectively. These results indicate that the Konya–Akşehir and Burdur–Bucak locations exhibited especially stronger antibacterial activity against Gram-negative bacteria.

Antibacterial activity was observed against *Staphylococcus aureus* in all species, but lower zone diameters were recorded compared to *E. coli*. Antibacterial effects were detected in the range of 7–8 mm at the Konya–Akşehir location, 7–8 mm at the Burdur–Bucak location, 14–23 mm at the Muğla–Köyceğiz location, and 7–8 mm at the Balıkesir–Sındırgı location. The activity at the Muğla–Köyceğiz location in the second year (23 mm) was determined to be significantly higher.

The microorganisms *Aspergillus niger*, *Penicillium roqforti*, and *Penicillium digitatum* were used to determine the antifungal activity of seed oils of *P. spina-christi* collected from different locations. The results show that the oils had no antifungal effect on these microorganisms.

The findings indicate that the zone-forming samples derived from *P. spina-christi* seed oils exhibited a bacteriostatic (growth-slowing/stopping) antibacterial effect, compared to the bactericidal (bacteria-killing) effect observed with antibiotic disks. In contrast, no inhibition zones were formed on the tested mold species (*A. niger*, *P. roqueforti*, *P. digitatum*). This suggests that although the components of the oil affected the bacterial cell wall permeability, they did not have a sufficient effect on the fungal cell structure. This disparity may be attributed to the existence of bioactive components within the oil, including tocopherols, phenolic compounds, and phytosterols. Phenolic compounds are known to inhibit bacterial growth by disrupting cell membrane integrity or by inhibiting enzyme systems [29,30].

Using the agar well diffusion method, the antimicrobial properties of water and ethanol extracts from the fruit and leaf sections of the *P. spina-christi* plant were investigated against the microorganisms *B. cereus*, *B. subtilis*, *E. coli*, *K. pneumoniae*, *P. multocida*, *P. aeruginosa*, *S. aureus*, *Y. enterocolitica*, and *C. albicans*. Ethanol extracts from the fruit and leaf sections showed the largest zone diameters among all microorganisms, with the exception of *P. multicoda* and *B.Cereus*, according to data from these antimicrobial investigations. Furthermore, the plant fruit’s ethanol extract demonstrated greater effectiveness against *P. aeruginosa*, *K. pneumoniae*, and *C. Albicans* [31]. Antimicrobial activity tests employing the well plate diffusion method were conducted on *P. mirabilis*, *S. sonnei*, *M. luteus*, *S. faecalis*, *S. aureus*, and *E. coli* using ethanolic extracts of various parts of plants (bark, fruit, flower, leaf, and root) of the *P. spina-christi* plant. The extracts demonstrated activity over all Gram-positive bacteria tested (*M. luteus*, *S. faecalis*, and *S. aureus*), forming distinct inhibition zones ranging from 8.5 to 14.0 mm in diameter. Root extracts showed the strongest action (inhibition zones between 10.0 and 14.0 mm). The extracts’ antibacterial activity against *S. sonnei* was measured at 8.5 to 10.0 mm in diameter. The other examined Gram-negative bacteria, *P. mirabilis* and *E. coli*, showed no signs of activity [32].

Based on these findings and literature comparisons, this investigation on the biological activity and chemical composition of *P. spina-christi*. seeds collected from four different regions of Türkiye shows that the species contains significant amounts of bioactive components. A non-toxicological level of HCN and 18 different phenolic compound compositions were observed in the seeds. High concentrations of α-tocopherol and total phenolic content were detected in the seed oils, contributing to their antioxidant activity. Mineral and sterol compositions analysis in the seed oils revealed that *P. spina-christi* seed oils are a plant-based oil source rich in both nutritional and functional contents, including diverse macrominerals (P, S, K, Ca, Mg, Na), microminerals (Si, Pt, Pd, Ge, Sn, etc.), and phytosterols (β-sitosterol, stigmasterol, etc.). In addition, bacteriostatic properties were detected in the seed oils. With the exception of antioxidant activity, sterol composition, and certain mineral contents, location and harvest year factors were found to have statistically significant effects on the chemical composition.

Overall, the results indicate that the phytochemical profiles of *P. spina-christi* seeds and seed oils are largely influenced by the collection site. Among the investigated regions, samples from Konya–Akşehir were generally characterized by higher seed phenolic content, HCN levels, and phenolic compound diversity, whereas samples from Muğla–Köyceğiz and Balıkesir–Sındırgı exhibited relatively higher α-tocopherol levels and distinct mineral profiles. These differences among the locations may be associated with variations in their climatic conditions, soil composition, and ecological characteristics.

Monitoring the locations for two years revealed that the harvest year plays a critical role in shaping the composition of both seeds and seed oils. While a general decrease in seed phenolic content and HCN levels was observed in the second harvest year, α-tocopherol concentrations in the seed oils increased markedly across all locations. This finding suggests that annual environmental conditions influence the accumulation of compounds in seeds and oils. The interaction between collection site and harvest year revealed that some locations exhibited relatively stable compositional profiles over both years (for example, protocatechuic acid in Burdur–Bucak and Balıkesir–Sındırgı; tannic acid in Muğla–Köyceğiz; As, Cr, Li, W, and Ti in Konya–Akşehir; Pb and Zr in Muğla–Köyceğiz; W in Balıkesir–Sındırgı; and K in Burdur–Bucak for mineral composition; and antioxidant activity in Konya–Akşehir and Burdur–Bucak), whereas others exhibited pronounced interannual fluctuations.

## 3. Materials and Methods

### 3.1. Plant Materials

*P. spina-christi* fruits analyzed in the current study were gathered from nature in October–November of 2023 and 2024 from four different regions of Türkiye (Marmara Region: Balıkesir–Sındırgı; Central Anatolia Region: Konya–Akşehir; Mediterranean Region: Burdur–Bucak; Aegean Region: Muğla–Köyceğiz), with the necessary permits obtained from the Ministry of Forestry and Agriculture of Türkiye (Table 11). The dried fruits obtained from each location (approximately 40–50 kg) were first crushed by passing them through a seed crushing machine after the collection process. The crushed fruit seeds were passed through sieves of different sizes, and the seeds were obtained by separating the shell pieces with the help of ventilation. The seeds, cleared of foreign materials (fruit peels, crumbs, etc.), were placed in clean cloth bags and kept in refrigerated conditions (+4–10 °C) for approximately 10 days. The seeds and oils *of P. spina-christi* obtained as a result of the processes were subjected to the following analyses.

### 3.2. Oil Extraction from Seeds

Hexane (Merck) (Merck KGaA, Darmstadt, Germany) with 98% purity and a semi-automatic oil determination device (Lab312, Calıskan Lab, Ankara, Türkiye) were used to extract oil from *P. spina-christi* seeds. Seed samples were ground prior to extraction. Approximately 5 g of each ground sample was weighed, tared, and placed on filter paper before being transferred to the sample section of the device. Extraction cups were filled with 50 mL of hexane (Merck, 98% purity) (Merck KGaA, Darmstadt, Germany) and placed in the device to collect the extracted oil. The extraction process involved boiling the samples at 90 °C for 50 min, followed by extraction at 85 °C for an additional 40 min. Finally, the valves were closed, and solvent recovery was conducted at 80 °C for 20 min, thus completing the oil extraction process [33]. Filter paper was used to filter the resultant oils (Whatman#1) (Whatman, UK), which were then transferred to dark-colored glass bottles, treated with nitrogen gas, and stored at room temperature (+22 °C).

### 3.3. Analyses Performed on Paliurus spina-christi Seeds

#### 3.3.1. Determination of HCN (Hydrocyanic Acid) Content in Seeds

A modified titrimetric approach based on the AOAC Method No: 915.03 (2000) standard analysis was used to measure the HCN content in *P. spina-christi* seeds. Approximately 20 g of *P. spina-christi* seed samples were placed in a Kjeldahl tube, followed by the addition of 200 mL of distilled water. The tube was then tightly sealed and kept at room temperature for four hours. Subsequently, it was placed in a distillation unit, and the distillate was collected in 20 milliliters of 2.5% NaOH solution (Merck). In a graduated cylinder, the obtained mixture was transferred to a final volume of 250 mL. Then, 8 mL of 6 N NH_4_OH (Merck) and 2 mL of 5% KI (Merck) solutions were added to a 100 mL aliquot transferred from the cylinder into an Erlenmeyer flask. The prepared solution was subsequently titrated against 0.02 M AgNO_3_ (Merck) [34].

#### 3.3.2. Phenolic Content in Seeds

The Folin–Ciocalteu analytical procedure was employed to quantify the total phenolic compounds present in the ground and dehydrated seed samples [35]. One gram of powdered seed samples was sonicated with 10 mL of acidified methanol (HCl:MeOH) (Merck) and subsequently incubated at 4 °C for 12 h. The resulting solution was filtered using filter paper (Whatman#1) and centrifuged for 10 min at 3500 rpm. Following separation of the extract, the remaining solid residue was re-extracted twice, each time with 5 mL of acidified methanol, using the same procedure. The extracts obtained from the three extraction steps were combined and thoroughly mixed. A 100 μL aliquot of the diluted solution was employed for the following analysis after a 0.5 mL portion of the combined extract was diluted 5 times. One milliliter of phenol reagent (Merck), 1 milliliter of 10% NaHCO_3_ (Merck), and 4 milliliters of distilled water were combined with this 100 microliter aliquot. Subsequently, the mixture was left in the dark for an hour. Plotting known gallic acid solutions against absorbance at 760 nm produced a calibration curve (R^2^ = 0.994) from which the total phenolic content was determined. The findings are expressed as milligrams of gallic acid equivalent (GAE) per gram of the samples’ fresh weight.

#### 3.3.3. LC-MS/MS Analysis of *Paliurus spina-christi* Seeds

**Figure 1:**
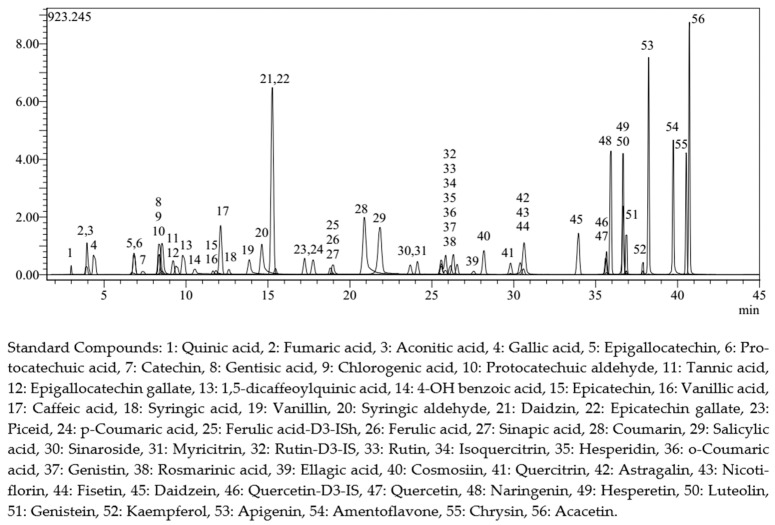
Standard mixture’s LC-MS/MS chromatograms [37].

Ten grams of each separate sample was removed employing the Soxhlet extraction technique with n-hexane (Merck) to analyze the phenolic content of *P. spina-christi* seeds. In an Erlenmeyer flask containing 100 mL of methanol, the defatted seed extracts were agitated for 8 h using a magnetic stirrer. Then, the resulting extract was filtered off, and the remaining seed samples underwent the same extraction procedure. All obtained extracts were combined, and a rotary evaporator operating at 45 °C was used to extract the methanol [36]. Quantitative analysis of 53 phytochemical compounds in the methanol extracts of *P. spina-christi* seed samples was performed employing a previously created and approved LC-MS/MS chromatographic technique. The standard chromatogram of phytochemical compounds is presented in Figure 1 [37]. The analyses were conducted using a Shimadzu-Nexera brand ultra-high-performance liquid chromatograph (UHPLC) (Shimadzu Corporation/Kyoto/Japan) coupled with a tandem mass spectrometer (Shimadzu Corporation/Kyoto/Japan). A column oven (CTO-10ASvp model, Shimadzu Corporation/Kyoto/Japan), an autosampler (SIL-30AC model, Shimadzu Corporation/Kyoto/Japan), a binary pump (LC-30AD type, Shimadzu Corporation/Kyoto/Japan), and a degasser (DGU-20A3R model, Shimadzu Corporation/Kyoto/Japan) make up this UHPLC system. Separation of the analytes was performed on a reversed-phase Agilent Poroshell 120 EC-C18 analytical column (Agilent Technologies, Germany) with dimensions of 150 mm × 2.1 mm and a particle size of 2.7 µm. The temperature of the column was kept at 40 °C. The elution gradient was composed of eluent A (water + 5 mM ammonium formate + 0.1% formic acid) and eluent B (methanol + 5 mM ammonium formate + 0.1% formic acid). The gradient elution profile was 20–100% B (0–25 min), 100% B (25–35 min), and 20% B (35–45 min). The solvent flow rate was set at 0.5 mL/min, while the injection volume was kept at 5 µL. A tandem mass spectrometer of the Shimadzu LCMS-8040 (Shimadzu Corporation/Kyoto/Japan) type was employed for mass spectrometric detection.

### 3.4. Analyses of Paliurus spina-christi Mill. Seed Oils

#### 3.4.1. Phenolic Content in Seed Oils

The Folin–Ciocalteu colorimetric technique was used to assess the total phenolic content in the *P. spina-christi* seed oil extracts [35]. For this assay, 300 mg of each oil sample was diluted in 1.5 mL of ethanol (Merck), and an ethanolic extract was obtained by sonicating the mixture three times for 5 min at 25 °C. Subsequently, cuvettes were filled with solution comprising 300 μL of Na_2_CO_3_ (20% *w*/*v*) (Merck), 40 μL of the ethanolic extract, 100 μL of Folin–Ciocalteu reagent (Merck), and 1.2 mL of distilled water. After 2 h of incubation at room temperature in a dark atmosphere, the cuvettes were tested for absorbance at 765 nm using a spectrophotometer (SOIF UV/VIS, Soif UV-5100B, Shanghai Metash Instruments Co., Ltd., Shanghai, China). A calibration curve was employed to determine the concentration of total phenolic content (R^2^ = 0.9977) generated from the absorbance values of gallic acid solutions at 765 nm. Milligrams of GAE per gram of oil were employed to express the results (mg GAE/g).

#### 3.4.2. Seed Oils Antioxidant Activity

A technique based on the DPPH (2,2-diphenyl-1-picrylhydrazyl) radical elimination assay developed by Brand-Williams et al. (1995) [38] was modified to assess the antioxidant activity of oils extracted from *P. spina-christi* seeds. For this purpose, 300 mg of seed oil sample was mixed in 1.5 mL of ethanol (Merck), and the resultant solution was then sonicated three times for five minutes at 25 °C. Following sonication, the ethanolic extract was collected, and a 1 mM DPPH stock solution was prepared. For analysis, cuvettes were filled with 3.8 mL of DPPH (6 × 10^−5^ M) solution and 0.2 mL of ethanolic extract. The prepared cuvettes were incubated at room temperature for half an hour in the dark. Using an SOIF UV/VIS spectrophotometer, absorbance measurements for the samples and reference groups were determined at a wavelength of 517 nm at the conclusion of the incubation period. Using Trolox (6-hydroxy-2,5,7,8-tetramethylchroman-2-carboxylic acid) as a reference antioxidant, the antioxidant activity of the seed oils was calculated using a standard calibration curve (R^2^ = 0.9997). Trolox equivalent value (TE) per gram of oil (mg TE/g) is used to express the findings of the triplicate analyses.

#### 3.4.3. α-Tocopherol Content in Seed Oils

α-tocopherol analysis of *P. spina-christi* seed oils was performed using a Shimadzu HPLC device with an RF-20A Fluorescence detector (Shimadzu Corporation/Kyoto/Japan) using a hexane/isopropyl alcohol (95/5) mobile phase [39]. The analysis time was 6 min, and 1 mL/min was accepted as the flow rate. The column used was Inert Sustain C18 (5 μm, 4.6 × 250 mm) (GL Sciences/Japan), and the temperature of the column was kept at 40 °C. The excitation and emission wavelengths for vitamin E detection were set at 295 nm and 330 nm, respectively. The main stock vitamin E was dissolved in hexane, and the necessary dilutions were prepared with the mobile phase. The calibration curve was prepared using concentrations of 5, 10, 25, and 50 ppm, and the R^2^ value of the calibration curve was determined as 0.998. During sample preparation, the samples were dissolved in the mobile phase, and appropriate dilutions were made to ensure that the results fell within the calibration curve range. The extracts’ α-tocopherol concentration was measured in milligrams per kilogram of oil.

#### 3.4.4. Seed Oils Sterol Compositions

Gas chromatography (GC) was used to assess the sterol content of the *P. spina-christi* seed oils in accordance with the technique specified by the International Olive Oil Council [40]. Sterol composition analyses were performed using a Shimadzu GC-2025 type gas chromatograph (Shimadzu Corporation/Kyoto/Japan). The operating conditions of the GC device were as follows: flame ionization detector (FID) (Shimadzu Corporation/Kyoto/Japan), N_2_ used as the carrier gas, split proportion of 250:0, flow rate of 0.80 mL/min, injecting block heat of 280 °C, sensor heat of 290 °C, column temperature of 260 °C, and injection volume of 1 μL.

#### 3.4.5. Seed Oils Mineral Composition

Ash was placed in a Milestone Ethos One microwave digestion system (Milestone Srl, Sorisole, Italy), and the seed oil sample was placed in a combustion tank. Ultrapure water was used to dilute the resultant solution to the required final volume. The samples’ mineral compositions were measured by comparing them to reference solutions with known concentrations. A Spectro inductively coupled plasma optical emission spectrometer (ICP-OES, Model: SpectroBlue) (SPECTRO Analytical Instruments GmbH, Kleve, Germany) was used for the measurements [41]. The ICP-OES’s operational parameters were as follows: Pump Speed (rpm): 30; Nebulizer Flow (L/min): 0.8; Auxiliary Gas Flow (L/min): 0.8; Quartz Plasma Torc; Coolant Flow (L/min): 13; Axial and Radial Plasma View Mode; Plasma Power (W): 1200; Spray Chamber: Cyclonic.

#### 3.4.6. Seed Oils Antimicrobial Activity Determination

##### Antibacterial Activity Determination

The agar diffusion technique was used to test the materials’ antibacterial properties [42]. For this purpose, ATCC25923 Gram-positive *Staphylococcus aureus* bacteria and ATCC25922 Gram-negative *Escherichia coli* were tested. The bacteria were incubated in 10 mL of nutrient broth (NB) in 15 mL glass tubes at 37 °C at a shaking speed of 120 rpm. Using sterile swab sticks, bacterial cells with 0.5 density (McFarland) were placed on Petri dishes containing Mueller Hinton Agar (MHA) and cultured for one day at 37 °C. For the application of the samples, 5 mm diameter wells were opened with a disk punch and 150 μL from each sample was transferred to the wells. Penicillin-G (Pen-G), ampicillin (Amp), and antibiotic disks containing amoxicillin (Amx) were utilized as positive controls. The study was repeated three times, and the average of the results obtained was taken. Since the structure of the oils was not suitable for the agar diffusion method, the well diffusion method was applied to the oils, and agar diffusion was applied to the antibiotic disks.

##### Antifungal Activity Determination

The antifungal properties of the seed oil samples were assessed against *Aspergillus niger* (BTU001), *Penicillium roqforti* (BTU003), and *Penicillium digitatum* (BTU004) using the agar well diffusion method. The fungal isolates were supplied from the culture collection of the Microbiology Laboratory, Faculty of Food Science and Technology, Bursa Technical University (Bursa, Türkiye) [43]. Fungal cultures were initially grown on Sabouraud Dextrose Agar (SDA) medium. Fungal spores harvested from the cultures were suspended in a solution containing 0.1% Tween 80 (7 mL). These suspensions were then serially diluted with physiological saline (FTS) (10^3^–10^6^) and inoculated onto Petri dishes containing SDA. Three wells, each 7 mm in diameter, were created in the agar. A volume of 30 µL of the prepared seed oils extracts was applied to every well, and the Petri dishes were cultured at 25–28 °C for one week. Following incubation, the diameters of the resulting inhibition zones were measured with a digital micrometer [44]. Additionally, 0.25 mg/mL amphotericin B was used as a positive control.

### 3.5. Statistical Analyses

Statistical analyses of the analytical measurements obtained were performed using the Minitab 21 [45] program. The analyses were performed on data obtained over two different years (2023–2024) from four different sampling groups, following a 4 × 2 × 2 × 13 experimental design with at least two replications. The data were first analyzed using one-way ANOVA and, if a statistically significant difference (*p* < 0.05) was detected, multiple comparisons were performed using Tukey’s test. Finally, the groups were lettered.

## 4. Conclusions

In conclusion, the results obtained demonstrate the functional food and phytotherapy potential of *P. spina-christi* seeds collected from different regions of Türkiye, particularly in terms of their hydrogen cyanide, total phenol content, and phenolic compound profile, as well as the α-tocopherol, sterol, and mineral composition and bacteriostatic properties of the seed oils, within the context of scientific contribution and practical application. Furthermore, the results provide preliminary data on the potential use of this plant as a food supplement, functional oil formulation, and natural preservative agent. It was also determined that *P. spina-christi* seeds and oils show potential for use in pharmaceutical, food, and vegetable oil technology due to their chemical, toxicological, phytochemical, and antimicrobial properties. On the other hand, the ecotype and year-based variations in the results obtained in our study indicate that the chemical and bioactive composition of *P. spina-christi* seeds and seed oils was largely influenced by both the collection site and the harvest year. While some parameters remained relatively stable (for example, protocatechuic acid in Burdur–Bucak and Balıkesir–Sındırgı; tannic acid in Muğla–Köyceğiz; As, Cr, Li, W, and Ti in Konya–Akşehir; Pb and Zr in Muğla–Köyceğiz; W in Balıkesir–Sındırgı; K in Burdur–Bucak for mineral composition; and antioxidant activity in Konya–Akşehir and Burdur–Bucak samples), others exhibited significant year-to-year variation, highlighting the importance of temporal monitoring. Based on the obtained data, it is recommended that further studies focus on standardization, bioavailability, and clinical validation to enable the use of *P. spina-christi* seeds and oils as human food. This will allow the *P. spina-christi* plant, particularly its seeds and oils, to be evaluated as potential products in the context of human nutrition, both as food supplements and in traditional medicine practices.

## Figures and Tables

**Table 1:** HCN contents of *Paliurus spina-christi* Mill. seeds.

Locations	1st Harvest Year (g/kg)	2nd Harvest Year (g/kg)	Average (g/kg)
Konya–Akşehir	1.87 ± 0.12 ^A^	0.99 ± 0.18 ^b^	1.43 ± 0.15 ^X^
Burdur–Bucak	1.37 ± 0.36 ^B^	1.29 ± 0.36 ^a^	1.33 ± 0.25 ^Y^
Muğla–Köyceğiz	1.24 ± 0.30 ^C^	0.99 ± 0.18 ^b^	1.11 ± 0.22 ^Z^
Balıkesir–Sındırgı	1.13 ± 0.06 ^D^	0.99 ± 0.06 ^b^	1.11 ± 0.06 ^Z^

±: Standard deviation; A–D: First harvest year difference between locations; a–b: second harvest year difference between locations; X–Z: harvest year mean differences; *p* < 0.05 was evaluated as the significance level.

**Table 2:** Total phenolic contents of *Paliurus spina-christi* Mill. seeds.

Locations	1st Harvest Year (mg GAE/g)	2nd Harvest Year (mg GAE/g)	Average (mg GAE/g)
Konya–Akşehir	17.52 ± 0.39 ^A^	13.44 ± 0.39 ^a^	15.48 ± 2.89 ^X^
Burdur–Bucak	6.10 ± 0.39 ^C^	7.59 ± 0.58 ^c^	6.84 ± 1.06 ^Z^
Muğla–Köyceğiz	9.22 ± 0.19 ^B^	7.05 ± 0.19 ^c^	8.14 ± 1.54 ^Z^
Balıkesir–Sındırgı	9.90 ± 0.00 ^B^	10.18 ± 0.39 ^b^	10.04 ± 0.19 ^Y^

±: Standard deviation; A–C: first harvest year difference between locations; a–c second harvest year difference between locations; X–Z: harvest year mean differences; *p* < 0.05 was evaluated as the significance level.

**Table 3:** Analytical parameters of the LC-MS/MS method and phenolic compounds and contents of *Paliurus spina-christi* Mill. seeds.

						Konya–Akşehir		Burdur–Bucak		Muğla–Köyceğiz		Balıkesir–Sındırgı	
No	Analytes	RT	M.I. (*m*/*z*)	F.I. (*m*/*z*)	U	1st Harvest Year	2nd Harvest Year	1st Harvest Year	2nd Harvest Year	1st Harvest Year	2nd Harvest Year	1st Harvest Year	2nd Harvest Year
1	Quinic acid	3.0	190.8	93.0	0.0372	0.655 ± 0.012 ^A^	0.725 ± 0.013 ^a^	0.192 ± 0.004 ^D^	0.168 ± 0.003 ^d^	0.633 ± 0.012 ^B^	0.269 ± 0.005 ^c^	0.489 ± 0.009 ^C^	0.455 ± 0.008 ^b^
2	Fumaric aid	3.9	115.2	40.9	0.0091	0.114 ± 0.001 ^A^	ND	ND	ND	ND	ND	ND	ND
3	Aconitic acid	4.0	172.8	129.0	0.0247	ND	ND	ND	ND	ND	ND	0.01	ND
4	Gallic acid	4.4	168.8	79.0	0.0112	0.131 ± 0.001 ^D^	0.169 ± 0.001 ^d^	0.192 ± 0.001 ^B^	0.232 ± 0.001 ^b^	0.146 ± 0.001 ^C^	0.238 ± 0.001 ^a^	0.329 ± 0.002 ^A^	0.210 ± 0.001 ^c^
5	Epigallocatechin	6.7	304.8	219.0	0.0184	ND	ND	ND	ND	ND	ND	ND	ND
6	Protocatechuic acid	6.8	152.8	108.0	0.0350	0.032 ± 0.001 ^A^	0.038 ± 0.001 ^a^	0.022 ± 0.000 ^D^	0.020 ± 0.000 ^c^	0.028 ± 0.000 ^C^	0.014 ± 0.000 ^d^	0.029 ± 0.001 ^B^	0.028 ± 0.000 ^b^
7	Catechin	7.4	288.8	203.1	0.0221	ND	ND	ND	ND	ND	ND	ND	ND
8	Gentisic acid	8.3	152.8	109.0	0.0167	ND	ND	ND	ND	ND	ND	ND	ND
9	Chlorogenic acid	8.4	353.0	85.0	0.0213	ND	ND	ND	0.014 ± 0.000 ^a^	ND	ND	ND	ND
10	Protocatechuic aldehyde	8.5	137.2	92.0	0.0396	ND	ND	ND	ND	ND	ND	ND	ND
11	Tannic acid	9.2	182.8	78.0	0.0190	0.348 ± 0.003 ^A^	0.052 ± 0.000 ^b^	0.032 ± 0.000 ^D^	0.056 ± 0.001 ^a^	0.046 ± 0.000 ^C^	0.048 ± 0.000 ^d^	0.078 ± 0.001 ^B^	0.050 ± 0.000 ^c^
12	Epigallocatechin gallate	9.4	457.0	305.1	0.0147	ND	ND	ND	ND	ND	ND	ND	ND
13	Cynarin	9.8	515.0	191.0	0.0306	ND	ND	ND	ND	ND	ND	ND	ND
14	*p*-OH benzoic acid	10.5	137.2	65.0	0.0237	ND	ND	ND	ND	ND	ND	ND	ND
15	Epicatechin	11.6	289.0	203.0	0.0221	ND	ND	ND	ND	ND	ND	ND	ND
16	Vanillic acid	11.8	166.8	108.0	0.0145	ND	ND	ND	ND	ND	ND	ND	ND
17	Caffeic acid	12.1	179.0	134.0	0.0152	ND	ND	ND	ND	ND	ND	ND	ND
18	Syringic acid	12.6	196.8	166.9	0.0129	ND	ND	ND	ND	ND	ND	ND	ND
19	Vanillin	13.9	153.1	125.0	0.0122	ND	ND	ND	ND	ND	ND	ND	ND
20	Syringic aldehyde	14.6	181.0	151.1	0.0215	ND	ND	ND	ND	ND	ND	ND	ND
21	Daidzin	15.2	417.1	199.0	0.0202	ND	ND	ND	ND	ND	ND	ND	ND
22	Epicatechin gallate	15.5	441.0	289.0	0.0229	ND	ND	ND	ND	ND	ND	ND	ND
23	Piceid	17.2	391.0	135/106.9	0.0199	ND	ND	ND	ND	ND	ND	ND	ND
24	*p*-Coumaric acid	17.8	163.0	93.0	0.0194	ND	ND	ND	ND	ND	ND	ND	ND
25	Ferulic acid- D3-IS *	18.8	196.2	152.1	0.0170	NA	NA	NA	NA	NA	NA	NA	NA
26	Ferulic acid	18.8	192.8	149.0	0.0181	ND	ND	ND	ND	ND	ND	ND	ND
27	Sinapic acid	18.9	222.8	193.0	0.0317	ND	ND	ND	ND	ND	ND	ND	ND
28	Coumarin	20.9	146.9	103.1	0.0383	ND	ND	ND	ND	ND	ND	ND	ND
29	Salicylic acid	21.8	137.2	65.0	0.0158	ND	0.019 ± 0.000 ^a^	ND	ND	ND	ND	ND	ND
30	Cyranoside	23.7	447.0	284.0	0.0366	ND	ND	ND	ND	ND	ND	ND	ND
31	Miquelianin	24.1	477.0	150.9	0.0220	ND	ND	ND	ND	ND	ND	ND	ND
32	Rutin-D3-IS *	25.5	612.2	304.1	NA	NA	NA	NA	NA	NA	NA	NA	NA
33	Rutin	25.6	608.9	301.0	0.0247	ND	0.053 ± 0.001 ^a^	ND	ND	ND	ND	ND	0.028 ± 0.000 ^b^
34	Isoquercitrin	25.6	463.0	271.0	0.0220	0.022 ± 0.000 ^A^	0.050 ± 0.0010 ^a^	ND	ND	ND	ND	ND	0.018 ± 0.000 ^b^
35	Hesperidin	25.8	611.2	449.0	0.0335	0.022 ± 0.000 ^A^	0.016 ± 0.000 ^a^	ND	ND	ND	ND	ND	ND
36	*o*-Coumaric acid	26.1	162.8	93.0	0.0147	ND	ND	ND	ND	ND	ND	ND	ND
37	Genistin	26.3	431.0	239.0	0.0083	0.024 ± 0.000 ^A^	0.0050 ± 0.000 ^a^	0.010 ± 0.000 ^B^	ND	ND	ND	ND	ND
38	Rosmarinic acid	26.6	359.0	197.0	0.0130	ND	ND	ND	ND	ND	ND	ND	ND
39	Ellagic acid	27.6	301.0	284.0	0.0364	ND	ND	ND	ND	ND	ND	ND	ND
40	Cosmosiin	28.2	431.0	269.0	0.0083	0.034 ± 0.000 ^C^	0.022 ± 0.000 ^d^	0.073 ± 0.000 ^B^	0.055 ± 0.000 ^b^	0.025 ± 0.000 ^D^	0.044 ± 0.000 ^c^	0.078 ± 0.000 ^A^	0.065 ± 0.000 ^a^
41	Quercitrin	29.8	447.0	301.0	0.0268	ND	ND	ND	ND	ND	ND	ND	ND
42	Astragalin	30.4	447.0	255.0	0.0114	ND	ND	ND	ND	ND	ND	ND	ND
43	Nicotiflorin	30.6	592.9	255.0/ 284.0	0.0108	ND	ND	ND	ND	ND	ND	ND	ND
44	Fisetin	30.6	285.0	163.0	0.0231	ND	ND	ND	ND	ND	ND	ND	ND
45	Daidzein	34.0	253.0	223.0	0.0370	0.008 ± 0.000 ^A^	ND	ND	ND	ND	ND	ND	ND
46	Quercetin- D3-IS *	35.6	304.0	275.9	NA	NA	NA	NA	NA	NA	NA	NA	NA
47	Quercetin	35.7	301.0	272.9	0.0175	ND	ND	ND	ND	ND	ND	ND	ND
48	Naringenin	35.9	270.9	119.0	0.0392	0.007 ± 0.000 ^A^	0.003 ± 0.000 ^a^	0.005 ± 0.000 ^B^	0.003 ± 0.000 ^a^	ND	ND	ND	ND
49	Hesperetin	36.7	301.0	136.0/ 286.0	0.0321	ND	ND	ND	ND	ND	ND	ND	ND
50	Luteolin	36.7	284.8	151.0/ 175.0	0.0313	0.0050 ± 0.000 ^A^	ND	ND	ND	ND	ND	ND	ND
51	Genistein	36.9	269.0	135.0	0.0337	ND	ND	ND	ND	ND	ND	ND	ND
52	Kaempferol	37.9	285.0	239.0	0.0212	ND	ND	ND	ND	ND	ND	ND	ND
53	Apigenin	38.2	268.8	151.0/ 149.0	0.0178	0.004 ± 0.000 ^A^	ND	ND	ND	ND	ND	ND	ND
54	Amentoflavone	39.7	537.0	417.0	0.0340	0.049 ± 0.001 ^A^	0.010 ± 0.000 ^a^	0.005 ± 0.000 ^B^	ND	ND	ND	ND	ND
55	Chrysin	40.5	252.8	145.0/ 119.0	0.0323	0.004 ± 0.000 ^A^	ND	ND	ND	ND	ND	ND	ND
56	Acacetin	40.7	283.0	239.0	0.0363	0.022 ± 0.000 ^A^	0.007 ± 0.000 ^a^	ND	ND	ND	ND	ND	ND

RT: retention time; MI (*m*/*z*): molecular ions of the standard analytes (*m*/*z* ratio); FI (*m*/*z*): fragment ions; U (%): percent relative uncertainty at 95% confidence level (k = 2); NA: not applicable; ND: not detected. ±: Standard deviation; A–D: first harvest year difference between locations; a–d: second harvest year difference between locations; *p* < 0.05 was evaluated as the significance level. * IS: internal standard. Compounds were determined as mg analyte/g extract.

**Table 4:** α-tocopherol contents of *Paliurus spina-christi* Mill. seed oils.

Locations	1st Harvest Year (mg/kg)	2nd Harvest Year (mg/kg)	Average (mg/kg)
Konya–Akşehir	1177.2 ± 3.10 ^C^	3698 ± 21.50 ^a^	2437.6 ± 178.25 ^X^
Burdur–Bucak	1262.8 ± 5.82 ^B^	3794 ± 20.65 ^a^	2528.4 ± 178.98 ^X^
Muğla–Köyceğiz	1320.5 ± 2.83 ^A^	3036.5 ± 2.33 ^b^	2178.5 ± 121.34 ^Y^
Balıkesir–Sındırgı	1285.6 ± 7.06 ^A^	3361.5 ± 42.78 ^b^	2323.6 ± 146.79 ^Y^

±: Standard deviation; A–C 1st harvest year difference between locations; a–b 2nd harvest year difference between locations; X–Y Harvest year mean differences; *p* < 0.05 was evaluated as the significance level.

**Table 5:** Total phenolic contents of *Paliurus spina-christi* Mill. seed oils.

Locations	1st Harvest Year (mg GAE/g)	2nd Harvest Year (mg GAE/g)	Average (mg GAE/g)
Konya–Akşehir	109.37 ± 8.85 ^B^	84.62 ± 4.45 ^b^	96.99 ± 17.50 ^Y^
Burdur–Bucak	146.09 ± 2.56 ^A^	84.01 ± 0.51 ^b^	115.05 ± 43.90 ^X^
Muğla–Köyceğiz	145.41 ± 6.50 ^A^	92.33 ± 0.72 ^b^	118.87 ± 37.53 ^X^
Balıkesir–Sındırgı	101.12 ± 11.10 ^B^	134.15 ± 1.64 ^a^	117.64 ± 23.36 ^X^

±: Standard deviation; A–B: first harvest year difference between locations; a–b: second harvest year difference between locations; X–Y: harvest year mean differences; *p* < 0.05 was evaluated as the significance level.

**Table 6:** Antioxidant activity of *Paliurus spina-christi* Mill. seed oils.

Locations	1st Harvest Year (mg TE/g)	2nd Harvest Year (mg TE/g)	Average (mg TE/g)
Konya–Akşehir	0.151 ± 0.001 ^A^	0.150 ± 0.000 ^a^	0.150 ± 0.001 ^X^
Burdur–Bucak	0.150 ± 0.001 ^AB^	0.149 ± 0.002 ^a^	0.149 ± 0.000 ^X^
Muğla–Köyceğiz	0.149 ± 0.002 ^B^	0.146 ± 0.002 ^b^	0.147± 0.002 ^Y^
Balıkesir–Sındırgı	0.146 ± 0.001 ^B^	0.148 ± 0.004 ^b^	0.147 ± 0.001 ^Y^

±: Standard deviation; A–B first harvest year difference between locations; a–b: second harvest year difference between locations; X–Y: harvest year mean differences; *p* < 0.05 was evaluated as the significance level.

**Table 7:** Sterol compositions of *Paliurus spina-christi* Mill. seed oils.

Sterol (%)	Locations	1st Harvest Year	2nd Harvest Year	Average
β-sitosterol	Konya–Akşehir	60.80 ± 1.02 ^C^	72.21 ± 1.33 ^a^	66.51 ± 8.07 ^X^
	Burdur–Bucak	66.24 ± 0.79 ^B^	64.10 ± 1.32 ^b^	65.17 ± 1.51 ^X^
	Muğla–Köyceğiz	64.11 ± 1.04 ^B^	57.11 ± 1.94 ^b^	61.51 ± 4.95 ^X^
	Balıkesir–Sındırgı	70.44 ± 0.76 ^A^	61.48 ± 2.62 ^b^	65.96 ± 6.34 ^X^
Brassicasterol	Konya–Akşehir	-	0.02 ± 0.01 ^a^	0.01 ± 0.01 ^X^
	Burdur–Bucak	-	0.01 ± 0.00 ^a^	0.01 ± 0.01 ^X^
	Muğla–Köyceğiz	-	0.02 ± 0.01 ^a^	0.01 ± 0.01 ^X^
	Balıkesir–Sındırgı	-	0.02 ± 0.01 ^a^	0.01 ± 0.01 ^X^
Campesterol	Konya–Akşehir	6.93 ± 0.32 ^B^	7.58 ± 0.18 ^a^	7.26 ± 0.46 ^X^
	Burdur–Bucak	7.88 ± 0.26 ^A^	6.53 ± 0.52 ^a^	7.20 ± 0.96 ^X^
	Muğla–Köyceğiz	7.31 ± 0.34 ^A^	6.06 ± 1.12 ^a^	6.69 ± 0.88 ^X^
	Balıkesir–Sındırgı	7.29 ± 0.24 ^A^	6.43 ± 0.18 ^a^	6.86 ± 0.61 ^X^
Δ5-Avenasterol	Konya–Akşehir	8.89 ± 0.24 ^A^	5.57 ± 0.21 ^b^	7.23 ± 2.35 ^X^
	Burdur–Bucak	8.03 ± 0.22 ^B^	5.74 ± 0.38 ^b^	6.89 ± 1.62 ^X^
	Muğla–Köyceğiz	7.94 ± 0.11 ^B^	7.68 ± 0.22 ^a^	7.81 ± 0.19 ^X^
	Balıkesir–Sındırgı	7.96 ± 0.38 ^B^	5.50 ± 0.11 ^b^	6.73 ± 1.74 ^X^
Δ7-Avenasterol	Konya–Akşehir	-	0.28 ± 0.01 ^b^	0.14 ± 0.20 ^X^
	Burdur–Bucak	0.18 ± 0.03 ^A^	0.20 ± 0.01 ^b^	0.19± 0.01 ^X^
	Muğla–Köyceğiz	-	0.45 ± 0.07 ^a^	0.23 ± 0.32 ^X^
	Balıkesir–Sındırgı	-	0.18 ± 0.02 ^b^	0.09 ± 0.12 ^X^
Kolesterol	Konya–Akşehir	-	0.22 ± 0.04 ^a^	0.11 ± 0.15 ^X^
	Burdur–Bucak	-	0.16 ± 0.01 ^a^	0.08 ± 0.11 ^X^
	Muğla–Köyceğiz	-	0.15 ± 0.03 ^a^	0.08 ± 0.11 ^X^
	Balıkesir–Sındırgı	-	0.16 ± 0.01 ^a^	0.08 ± 0.11 ^X^
Stigmasterol	Konya–Akşehir	11.51 ± 0.65 ^B^	9.91 ± 0.10 ^a^	10.71 ± 1.13 ^X^
	Burdur–Bucak	14.30 ± 0.35 ^A^	9.09 ± 0.11 ^b^	11.70 ± 3.68 ^X^
	Muğla–Köyceğiz	11.98 ± 0.40 ^B^	7.75 ± 0.01 ^c^	9.86 ± 2.99 ^X^
	Balıkesir–Sındırgı	12.60 ± 0.40 ^B^	8.16 ± 0.25 ^c^	10.38 ± 3.14 ^X^
Δ7-Stigmastenol	Konya–Akşehir	-	0.21 ± 0.02 ^a^	0.10 ± 0.14 ^X^
	Burdur–Bucak	0.17 ± 0.06 ^A^	0.32 ± 0.00 ^a^	0.25 ± 0.11 ^X^
	Muğla–Köyceğiz	-	0.24 ± 0.01 ^a^	0.12 ± 0.17 ^X^
	Balıkesir–Sındırgı	-	0.36 ± 0.08 ^a^	0.18 ± 0.25 ^X^

±: Standard deviation; A–C: First harvest year difference between locations; a–c: second harvest year difference between locations; X: harvest year mean differences; *p* < 0.05 was evaluated as the significance level, β: beta, Δ: delta.

**Table 8:** Macromineral composition *Paliurus spina-christi* Mill. seed oils.

Minerals (ppm)	Locations	1st Harvest Year	2nd Harvest Year	Average
Mg	Konya–Akşehir	19.05 ± 0.72 ^D^	34.73 ± 0.97 ^a^	26.89 ± 8.62 ^X^
	Burdur–Bucak	25.35 ± 0.24 ^C^	27.72 ± 0.15 ^b^	26.53 ± 1.31 ^X^
	Muğla–Köyceğiz	42.76 ± 0.32 ^B^	21.33 ± 0.15 ^d^	32.04 ± 11.74 ^X^
	Balıkesir–Sındırgı	49.10 ± 0.23 ^A^	22.87 ± 0.16 ^c^	35.99 ± 14.37 ^X^
K	Konya–Akşehir	63.33 ± 2.40 ^A^	58.04 ± 0.90 ^a^	60.69 ± 3.32 ^X^
	Burdur–Bucak	55.76 ± 1.39 ^B^	55.38 ± 1.92 ^a^	55.57 ± 1.52 ^X^
	Muğla–Köyceğiz	54.42 ± 3.35 ^B^	56.14 ± 3.66 ^a^	55.28 ± 3.28 ^X^
	Balıkesir–Sındırgı	65.11 ± 1.08 ^A^	56.48 ± 1.01 ^a^	60.79 ± 4.82 ^X^
Na	Konya–Akşehir	33.58 ± 0.57 ^A^	29.47 ± 0.31 ^c^	31.52 ± 2.29 ^X^
	Burdur–Bucak	29.87 ± 1.27 ^B^	50.38 ± 0.24 ^a^	40.12 ± 11.26 ^X^
	Muğla–Köyceğiz	32.50 ± 0.30 ^A^	23.72 ± 0.59 ^d^	28.11 ± 4.83 ^Y^
	Balıkesir–Sındırgı	30.98 ± 0.33 ^B^	35.88 ± 0.58 ^b^	33.43 ± 2.72 ^X^
Ca	Konya–Akşehir	38.97 ± 0.70 ^D^	58.35 ± 0.41 ^b^	48.66 ± 10.63 ^X^
	Burdur–Bucak	51.07 ± 0.18 ^C^	61.24 ± 0.34 ^a^	56.15 ± 5.57 ^X^
	Muğla–Köyceğiz	92.49 ± 1.05 ^A^	40.40 ± 0.48 ^c^	66.44 ± 28.54 ^X^
	Balıkesir–Sındırgı	77.78 ± 0.81 ^B^	35.83 ± 0.12 ^d^	56.80 ± 22.98 ^X^
P	Konya–Akşehir	312.75 ± 0.00 ^D^	352.42 ± 0.14 ^a^	332.58 ± 21.73 ^X^
	Burdur–Bucak	324.75 ± 0.66 ^C^	310.08 ± 0.72 ^c^	317.42 ± 8.06 ^Y^
	Muğla–Köyceğiz	331.08 ± 0.80 ^B^	304.83 ± 0.76 ^d^	317.96 ± 14.39 ^Y^
	Balıkesir–Sındırgı	388.25 ± 0.43 ^A^	321.92 ± 0.52 ^b^	355.08 ± 36.33 ^X^
S	Konya–Akşehir	329.92 ± 0.14 ^D^	339.42 ± 0.29 ^b^	334.67 ± 5.21 ^Y^
	Burdur–Bucak	341.25 ± 0.43 ^B^	354.58 ± 0.14 ^a^	347.92 ± 7.31 ^X^
	Muğla–Köyceğiz	333.25 ± 0.25 ^C^	329.08 ± 0.14 ^d^	331.17 ± 2.29 ^Y^
	Balıkesir–Sındırgı	347.25 ± 0.43 ^A^	337.25 ± 0.66 ^c^	342.25 ± 5.50 ^X^

±: Standard deviation; A–D: first harvest year difference between locations; a–d: second harvest year difference between locations; X–Y: harvest year mean differences; *p* < 0.05 was evaluated as the significance level.

**Table 9:** Micromineral composition in *Paliurus spina-christi* Mill. seed oils.

Locations	Konya–Akşehir			Burdur–Bucak			Muğla–Köyceğiz			Balıkesir–Sındırgı		
Minerals (ppm)	1st Harvest Year	2nd Harvest Year	Average	1st Harvest Year	2nd Harvest Year	Average	1st Harvest Year	2nd Harvest Year	Average	1st Harvest Year	2nd Harvest Year	Average
Al	-	0.09 ± 0.06 ^c^	0.09 ± 0.06 ^Z^	1.48 ± 0.22 ^A^	0.48 ± 0.04 ^b^	0.98 ± 0.56 ^Y^	0.90 ± 0.10 ^B^	-	0.90 ± 0.10 ^Y^	1.61 ± 0.02 ^A^	1.95 ± 0.06 ^a^	1.78 ± 0.19 ^X^
As	10.19 ± 0.57 ^B^	10.43 ± 0.09 ^c^	10.31 ± 0.39 ^Y^	9.25 ± 0.16 ^C^	13.00 ± 0.28 ^b^	11.12 ± 2.06 ^Y^	12.76 ± 0.13 ^A^	14.14 ± 0.53 ^a^	13.45 ± 0.83 ^X^	9.10 ± 0.27 ^C^	11.18 ± 0.24 ^c^	10.14 ± 1.16 ^Y^
Ba	0.38 ± 0.02 ^C^	0.54 ± 0.02 ^b^	0.46 ± 0.09 ^Y^	0.37 ± 0.01 ^C^	0.37 ± 0.02 ^c^	0.37 ± 0.01 ^Y^	0.48 ± 0.01 ^B^	0.006 ± 0.042 ^d^	0.24 ± 0.33 ^Y^	0.69 ± 0.02 ^A^	1.49 ± 0.00 ^a^	1.09 ± 0.44 ^X^
Bi	5.57 ± 0.14 ^C^	6.88 ± 0.04 ^b^	6.23 ± 0.72 ^Y^	6.77 ± 0.05 ^B^	7.16 ± 0.03 ^a^	6.96 ± 0.22 ^X^	7.43 ± 0.06 ^A^	7.33 ± 0.26 ^a^	7.38 ± 0.18 ^X^	6.85 ± 0.06 ^B^	7.11 ± 0.11 ^a^	6.98 ± 0.16 ^X^
B	8.86 ± 1.13 ^C^	17.10 ± 0.49 ^b^	12.98 ± 4.58 ^Y^	21.76 ± 0.14 ^A^	19.02 ± 0.37 ^a^	20.39 ± 1.52 ^X^	18.44 ± 0.39 ^B^	11.71 ± 0.79 ^c^	15.07 ± 3.72 ^Y^	8.92 ± 0.53 ^C^	7.05 ± 0.51 ^d^	7.98 ± 1.12 ^Z^
Cd	1.34 ± 0.06 ^C^	1.55 ± 0.00 ^a^	1.44 ± 0.12 ^Y^	1.48 ± 0.03 ^B^	1.53 ± 0.01 ^a^	1.51 ± 0.03 ^X^	1.58 ± 0.00 ^A^	1.55 ± 0.02 ^a^	1.56 ± 0.03 ^X^	1.46 ± 0.01 ^B^	1.53 ± 0.03 ^a^	1.49 ± 0.04 ^X^
Cr	12.72 ± 1.07 ^D^	12.72 ± 0.36 ^a^	12.72 ± 0.71 ^Y^	15.46 ± 0.48 ^C^	14.16 ± 0.27 ^a^	14.81 ± 0.79 ^X^	19.71 ± 0.18 ^B^	9.81 ± 1.23 ^b^	14.76 ± 5.48 ^X^	26.38 ± 0.47 ^A^	14.19 ± 0.55 ^a^	20.28 ± 6.69 ^X^
Co	0.24 ± 0.02 ^D^	0.30 ± 0.02 ^b^	0.27 ± 0.04 ^Z^	0.34 ± 0.03 ^C^	0.41 ± 0.01 ^a^	0.37 ± 0.05 ^Y^	0.49 ± 0.02 ^A^	0.41 ± 0.03 ^a^	0.45 ± 0.05 ^X^	0.40 ± 0.02 ^B^	0.39 ± 0.02 ^a^	0.39 ± 0.02 ^X^
Cu	-	-	-	-	-	-	-	-	-	-	-	-
Ga	29.92 ± 1.77 ^B^	28.78 ± 0.28 ^b^	29.35 ± 1.29 ^Y^	34.86 ± 1.98 ^A^	32.36 ± 0.39 ^a^	33.61 ± 1.87 ^X^	33.76 ± 0.44 ^A^	32.27 ± 1.35 ^a^	33.01 ± 1.21 ^X^	36.10 ± 0.56 ^A^	32.00 ± 1.23 ^a^	34.05 ± 2.40 ^X^
In	7.48 ± 0.25 ^C^	8.87 ± 0.21 ^a^	8.18 ± 0.79 ^Y^	8.84 ± 0.18 ^B^	9.57 ± 0.09 ^a^	9.20 ± 0.42 ^X^	9.99 ± 0.20 ^A^	9.58 ± 0.50 ^a^	9.78 ± 0.41 ^X^	9.01 ± 0.30 ^B^	9.43 ± 0.17 ^a^	9.22 ± 0.31 ^X^
Fe	4.24 ± 0.15 ^D^	3.55 ± 0.24 ^c^	3.90 ± 0.42 ^Y^	6.36 ± 0.43 ^C^	4.12 ± 0.06 ^c^	5.24 ± 1.26 ^Y^	9.27 ± 0.05 ^B^	9.95 ± 0.15 ^b^	9.61 ± 0.39 ^Y^	14.15 ± 0.31 ^A^	31.70 ± 1.48 ^a^	22.92 ± 9.66 ^X^
Pb	8.64 ± 0.29 ^C^	10.28 ± 0.04 ^b^	9.46 ± 0.92 ^Y^	9.77 ± 0.11 ^B^	10.61 ± 0.06 ^a^	10.19 ± 0.47 ^X^	10.86 ± 0.10 ^A^	10.86 ± 0.22 ^a^	10.86 ± 0.15 ^X^	9.83 ± 0.09 ^B^	10.41 ± 0.12 ^b^	10.12 ± 0.33 ^X^
Li	2.39 ± 0.07 ^B^	2.36 ± 0.05 ^a^	2.38 ± 0.06 ^Y^	3.05 ± 0.10 ^A^	2.87 ± 0.18 ^a^	2.96 ± 0.16 ^X^	2.95 ± 0.14 ^A^	2.65 ± 0.34 ^a^	2.80 ± 0.28 ^X^	3.14 ± 0.20 ^A^	2.81 ± 0.34 ^a^	2.97 ± 0.31 ^X^
Mn	0.17 ± 0.01 ^D^	0.37 ± 0.01 ^a^	0.27 ± 0.11 ^Y^	0.32 ± 0.01 ^C^	0.33 ± 0.01 ^b^	0.33 ± 0.01 ^Y^	1.01 ± 0.01 ^A^	0.35 ± 0.01 ^a^	0.68 ± 0.36 ^X^	0.50 ± 0.01 ^B^	0.31 ± 0.01 ^b^	0.41 ± 0.11 ^X^
Ni	6.69 ± 0.29 ^C^	8.50 ± 0.04 ^b^	7.59 ± 1.01 ^Y^	8.02 ± 0.05 ^B^	8.56 ± 0.09 ^b^	8.29 ± 0.30 ^X^	9.15 ± 0.04 ^A^	8.84 ± 0.13 ^a^	8.99 ± 0.19 ^X^	8.36 ± 0.03 ^B^	8.21 ± 0.03 ^c^	8.28 ± 0.08 ^X^
Se	9.57 ± 0.45 ^B^	10.83 ± 0.24 ^b^	10.20 ± 0.76 ^Y^	9.94 ± 0.33 ^B^	11.68 ± 0.05 ^a^	10.81 ± 0.97 ^Y^	11.88 ± 0.15 ^A^	12.01 ± 0.19 ^a^	11.95 ± 0.17 ^X^	9.88 ± 0.22 ^B^	10.77 ± 0.10 ^b^	10.32 ± 0.51 ^Y^
Si	248.15 ± 1.14 ^A^	235.07 ± 3.63 ^a^	241.61 ± 7.56 ^X^	236.51 ± 1.15 ^B^	215.22 ± 1.49 ^b^	225.86 ± 11.72 ^X^	221.13 ± 1.00 ^C^	194.89 ± 1.35 ^c^	208.01 ± 14.41 ^Y^	214.21 ± 1.27 ^D^	186.80 ± 1.19 ^d^	200.51 ± 15.05 ^Y^
Ag	5.95 ± 0.27 ^B^	5.68 ± 0.31 ^a^	5.81 ± 0.30 ^Y^	6.59 ± 0.39 ^A^	6.18 ± 0.18 ^a^	6.38 ± 0.35 ^X^	6.54 ± 0.16 ^A^	6.14 ± 0.38 ^a^	6.34 ± 0.34 ^X^	6.87 ± 0.12 ^A^	6.19 ± 0.26 ^a^	6.53 ± 0.41 ^X^
Sr	0.13 ± 0.01 ^D^	0.23 ± 0.01 ^b^	0.18 ± 0.06 ^X^	0.28 ± 0.01 ^C^	0.55 ± 0.00 ^a^	0.41 ± 0.15 ^X^	0.49 ± 0.01 ^B^	0.16 ± 0.00 ^c^	0.33 ± 0.18 ^X^	0.56 ± 0.01 ^A^	0.17 ± 0.01 ^c^	0.37 ± 0.22 ^X^
Tl	15.90 ± 0.34 ^B^	16.67 ± 0.18 ^d^	16.29 ± 0.48 ^Y^	15.65 ± 0.05 ^B^	19.14 ± 0.28 ^b^	17.40 ± 1.92 ^Y^	18.98 ± 0.21 ^A^	20.40 ± 0.47 ^a^	19.69 ± 0.84 ^X^	15.41 ± 0.09 ^B^	17.46 ± 0.15 ^c^	16.44 ± 1.12 ^Y^
V	8.61 ± 0.46 ^A^	8.45 ± 0.38 ^b^	8.53 ± 0.39 ^Y^	9.41 ± 0.85 ^A^	9.77 ± 0.28 ^a^	9.59 ± 0.60 ^X^	9.23 ± 0.55 ^A^	9.10 ± 0.24 ^a^	9.17 ± 0.38 ^X^	10.09 ± 0.72 ^A^	8.79 ± 0.35 ^b^	9.44 ± 0.88 ^X^
Zn	0.20 ± 0.01 ^C^	0.35 ± 0.01 ^c^	0.28 ± 0.08 ^Y^	1.04 ± 0.04 ^B^	1.58 ± 0.04 ^a^	1.31 ± 0.30 ^X^	1.17 ± 0.01 ^A^	0.12 ± 0.01 ^d^	0.64 ± 0.57 ^Y^	1.14 ± 0.01 ^A^	0.48 ± 0.01 ^b^	0.81 ± 0.37 ^X^
Au	3.15 ± 0.13 ^B^	2.82 ± 0.02 ^b^	2.99 ± 0.20 ^Y^	3.27 ± 0.11 ^A^	3.17 ± 0.09 ^a^	3.22 ± 0.11 ^X^	3.27 ± 0.05 ^A^	2.99 ± 0.18 ^a^	3.13 ± 0.20 ^X^	3.47 ± 0.04 ^A^	3.26 ± 0.05 ^a^	3.37 ± 0.12 ^X^
Ge	40.09 ± 3.34 ^A^	37.30 ± 1.53 ^a^	38.70 ± 2.78 ^X^	32.19 ± 3.33 ^B^	45.03 ± 2.45 ^a^	38.61 ± 7.50 ^X^	43.03 ± 2.69 ^A^	44.66 ± 4.57 ^a^	43.85 ± 3.47 ^X^	30.53 ± 2.60 ^B^	38.74 ± 4.68 ^a^	34.63 ± 5.63 ^Y^
Mo	9.69 ± 0.33 ^B^	10.99 ± 0.03 ^c^	10.34 ± 0.75 ^Y^	10.15 ± 0.13 ^B^	40.45 ± 0.58 ^a^	25.30 ± 16.60 ^X^	11.69 ± 0.15 ^A^	12.01 ± 0.21 ^b^	11.85 ± 0.24 ^Y^	10.14 ± 0.11 ^B^	11.01 ± 0.04 ^c^	10.57 ± 0.49 ^Y^
Nb	17.97 ± 1.19 ^A^	16.87 ± 0.40 ^b^	17.42 ± 1.00 ^Y^	19.80 ± 1.49 ^A^	18.80 ± 0.29 ^a^	19.30 ± 1.11 ^X^	19.60 ± 0.07 ^A^	18.30 ± 0.61 ^a^	18.95 ± 0.81 ^X^	20.30 ± 0.02 ^A^	18.22 ± 0.52 ^a^	19.26 ± 1.19 ^X^
Pd	37.98 ± 1.22 ^B^	36.54 ± 0.16 ^b^	37.26 ± 1.11 ^Y^	42.62 ± 2.44 ^A^	39.82 ± 1.01 ^a^	41.22 ± 2.27 ^X^	40.58 ± 1.64 ^A^	39.25 ± 1.00 ^a^	39.91 ± 1.41 ^X^	44.13 ± 1.50 ^A^	39.62 ± 0.99 ^a^	41.87 ± 2.72 ^X^
Pt	71.76 ± 2.94 ^C^	86.32 ± 0.61 ^b^	79.04 ± 8.20 ^Y^	80.51 ± 1.59 ^B^	86.96 ± 1.00 ^a^	83.74 ± 3.73 ^X^	87.83 ± 1.05 ^A^	88.79 ± 1.11 ^a^	88.31 ± 1.10 ^X^	81.48 ± 0.96 ^B^	83.98 ± 0.61 ^c^	82.73 ± 1.55 ^X^
Sb	10.85 ± 0.61 ^B^	12.10 ± 0.11 ^c^	11.48 ± 0.79 ^Y^	11.48 ± 0.90 ^B^	21.49 ± 0.73 ^a^	16.49 ± 5.53 ^X^	14.02 ± 0.27 ^A^	14.84 ± 0.50 ^b^	14.43 ± 0.57 ^X^	11.72 ± 0.13 ^B^	12.59 ± 0.23 ^c^	12.16 ± 0.50 ^X^
Sn	29.40 ± 1.62 ^B^	30.88 ± 0.27 ^c^	30.14 ± 1.31 ^Y^	28.30 ± 0.05 ^B^	35.94 ± 0.46 ^b^	32.12 ± 4.20 ^Y^	35.41 ± 0.34 ^A^	38.90 ± 1.23 ^a^	37.16 ± 2.08 ^X^	27.68 ± 0.16 ^B^	32.62 ± 0.54 ^c^	30.15 ± 2.73 ^Y^
W	13.07 ± 0.11 ^C^	13.00 ± 0.10 ^b^	13.03 ± 0.10 ^Z^	13.87 ± 0.31 ^B^	14.17 ± 0.10 ^a^	14.02 ± 0.26 ^Y^	14.66 ± 0.07 ^A^	13.83 ± 0.53 ^a^	14.24 ± 0.57 ^X^	14.70 ± 0.27 ^A^	14.48 ± 0.20 ^a^	14.59 ± 0.24 ^X^
Ti	4.73 ± 0.14 ^A^	4.72 ± 0.55 ^a^	4.73 ± 0.36 ^X^	5.36 ± 0.45 ^A^	4.38 ± 0.27 ^a^	4.87 ± 0.63 ^X^	4.94 ± 0.15 ^A^	4.48 ± 0.15 ^a^	4.71 ± 0.29 ^X^	5.34 ± 0.43 ^A^	5.15 ± 0.09 ^a^	5.24 ± 0.29 ^X^
Zr	5.85 ± 0.14 ^B^	5.65 ± 0.09 ^a^	5.75 ± 0.15 ^Y^	6.53 ± 0.21 ^A^	6.18 ± 0.08 ^a^	6.35 ± 0.24 ^X^	6.19 ± 0.12 ^B^	6.11 ± 0.29 ^a^	6.15 ± 0.21 ^X^	6.69 ± 0.07 ^A^	6.15 ± 0.27 ^a^	6.42 ± 0.34 ^X^

±: Standard deviation; A–D: first harvest year difference between locations; a–d: second harvest year difference between locations; X–Z: harvest year mean differences; *p* < 0.05 was evaluated as the significance level.

**Table 10:** Antimicrobial activities of *Paliurus spina-christi* Mill. seed oils.

Locations	Harvest Year	*E. coli* (mm)	*S. aureus* (mm)
Konya–Akşehir	1st Harvest Year	36 ± 1.0 ^A^	7 ± 0.5 ^C^
	2nd Harvest Year	10 ± 0.3 ^d^	8 ± 0.6 ^b^
	Average	23 ± 18.38 ^Y^	7.5 ± 0.71 ^Y^
Burdur–Bucak	1st Harvest Year	32 ± 2.0 ^B^	8 ± 0.3 ^B^
	2nd Harvest Year	23 ± 1.0 ^b^	7 ± 0.1 ^c^
	Average	27.5 ± 6.36 ^X^	7.5 ± 0.71 ^Y^
Muğla–Köyceğiz	1st Harvest Year	14 ± 0.8 ^D^	14 ± 1.0 ^A^
	2nd Harvest Year	22 ± 1.0 ^c^	23 ± 0,7 ^a^
	Average	18 ± 5.66 ^Z^	18.5 ± 6.36 ^X^
Balıkesir–Sındırgı	1st Harvest Year	28 ± 2.0 ^C^	8 ± 0.1 ^B^
	2nd Harvest Year	24 ± 0.7 ^a^	7 ± 0.6 ^c^
	Average	26 ± 2.83 ^X^	7.5 ± 0.71 ^Y^
Antibiotic Disks	Amx	27	27
	Amp	23	23
	Pen-G	12	13

mm: Inhibition zone diameter. ±: Standard deviation; A–D: first harvest year difference between locations; a–d: second harvest year difference between locations; X–Z: harvest year mean differences; *p* < 0.05 was evaluated as the significance level.

**Table 11:** Locations and harvest times of *Paliurus spina-christi* Mill. plant.

Locations	Collected Location	Harvest Time (Month/Year)
Konya–Akşehir	38°20′20.5″ N/31°25′24.8″ E/Konya/Türkiye	October–November/2023–2024
Burdur–Bucak	37°18′40.2″ N/30°30′16.2″ E/Burdur/Türkiye	October–November/2023–2024
Muğla–Köyceğiz	36°52′05.2″ N/28°36′33.3″ E/Muğla/Türkiye	October–November/2023–2024
Balıkesir–Sındırgı	39°15′13.7″ N/28°03′02.5″ E/Balıkesir/Türkiye	October–November/2023–2024

## Data Availability

The original contributions presented in this study are included in the article. Further inquiries can be directed to the corresponding author.

## Description

The article is based on a section of the first author Rabiya Safiye Çelebi’s ongoing and unpublished PhD thesis.
